# Regulation of the MIE Locus During HCMV Latency and Reactivation

**DOI:** 10.3390/pathogens9110869

**Published:** 2020-10-23

**Authors:** Abigail L. Dooley, Christine M. O’Connor

**Affiliations:** 1 Genomic Medicine, Lerner Research Institute, Cleveland Clinic, Cleveland, OH 44105, USA; dooleya2@ccf.org; 2 Cleveland Clinic Lerner College of Medicine of Case Western Reserve University, Cleveland Clinic, Cleveland, OH 44195, USA

**Keywords:** cytomegalovirus, MIEP, latency, reactivation, CMV

## Abstract

Human cytomegalovirus (HCMV) is a ubiquitous herpesviral pathogen that results in life-long infection. HCMV maintains a latent or quiescent infection in hematopoietic cells, which is broadly defined by transcriptional silencing and the absence of de novo virion production. However, upon cell differentiation coupled with immune dysfunction, the virus can reactivate, which leads to lytic replication in a variety of cell and tissue types. One of the mechanisms controlling the balance between latency and reactivation/lytic replication is the regulation of the major immediate-early (MIE) locus. This enhancer/promoter region is complex, and it is regulated by chromatinization and associated factors, as well as a variety of transcription factors. Herein, we discuss these factors and how they influence the MIE locus, which ultimately impacts the phase of HCMV infection.

## 1. Introduction

Human cytomegalovirus (HCMV) is a ubiquitous pathogen that infects 40–60% of the population in developed countries and up to 100% in underdeveloped nations [1]. Like all herpesviruses, HCMV establishes a lifelong infection in individuals and can reactivate from latency sporadically throughout one’s lifetime. In healthy individuals, the infection can result in either asymptomatic or mild symptoms in response to host innate and adaptive immune responses. Primary infection in immuno-competent children and adults rarely causes the disease; rather it is primary infection or reactivation of latent infection in severely immunocompromised and immunosuppressed individuals that pose a significant risk, which can cause serious morbidity and mortality. Additionally, congenital CMV (cCMV) infection in the immunonaïve, resulting from primary infection of the fetus, has an estimated incidence of 0.6–0.7% and 1–5% of all live births in developed and underdeveloped countries, respectively, making it the most common congenital infection worldwide [2]. For immunosuppressed individuals, including solid organ transplant (SOT) recipients, viral reactivation is particularly problematic. Symptomatic HCMV infection occurs in 8–32% of kidney, 22–29% of liver, 39–41% of lung/heart–lung, and almost 50% of pancreas transplant recipients [3]. The greatest risk factor for HCMV-associated disease in these patients is the serostatus mismatch between the donor and recipient (i.e., donor positive recipient negative [D+/R−] transplant) [4]. For example, in lung transplant recipients who received antiviral prophylaxis for 6 to 12 months, the incidence of HCMV-associated disease was 14.9%, with a higher incidence (26.6%) in D+/R− group [5]. In SOT, HCMV is also linked to a greater risk of graft rejection [6]. Additionally, HCMV remains a significant problem for individuals undergoing bone marrow or hematopoietic stem cell transplant (BMT or HSCT, respectively). The rate of HCMV reactivation following HSCT is 30–70% and is associated with a higher non-relapse mortality rate (relative risk [RR], 1.61 to 1.95) [7,8,9,10,11,12]. Mortality related to fatal HCMV-associated disease is still as high as 45–60% in HSCT recipients. CMV pneumonia and encephalitis are particularly fatal, despite aggressive treatments using antiviral agents and adjunctive therapies [13,14,15,16,17,18], which are accompanied by severe toxicity and viral resistance [19], highlighting the burden caused by this significant human pathogen. 

Currently, there are five antiviral therapies approved for the treatment of HCMV-associated disease by the FDA, including ganciclovir, valganciclovir, foscarnet, cidofovir, maribavir, and letermovir. In the transplant setting, there are two therapeutic strategies to combat HCMV-associated disease: (1) prophylaxis, where the drug is given to the patient from the time of transplant for a specific, pre-determined treatment course following the transplant; or (2) preemptive therapy, where patients are first monitored for HCMV infection and treated once they reach a detectable level of viremia [19]. Guidelines for treatment strategies are determined by transplant centers and depend on prior diagnoses and type of transplantation. First-line therapy for HCMV viremia is ganciclovir, an acyclovir prodrug that when activated by the viral kinase, UL97, serves as a chain terminator for viral DNA elongation due to its higher affinity for the viral polymerase [20]. Valganciclovir, the L-valyl ester prodrug of ganciclovir, is delivered orally, thereby increasing absorption and bioavailability, which can be up to 10-fold higher than that of oral ganciclovir. While effective, these first-line therapies often lead to viral resistance, most commonly manifested by viral mutations of the UL54 DNA polymerase or UL97 kinase genes [21], thereby necessitating second-line treatments cidofovir and foscarnet, both of which target UL54 [22] are administered in such cases, though their usage is restricted due to nephrotoxicity [23]. The FDA has bestowed Orphan Drug and Breakthrough Therapy Designations on maribavir for viremic/high-risk patients and transplant patients who are resistant or refractory to other treatments, respectively [24,25]. Although not approved in all settings, letermovir, a viral terminase complex (UL56) inhibitor which functions to inhibit the processing of viral progeny, is the newest FDA-approved drug for HCMV prophylaxis for seropositive HSCT recipients [23], who are at increased risk for reactivation [7]. As with other antiviral compounds, letermovir also leads to viral resistance, conferred by UL56 mutations, though these mutations do not impact viral fitness (e.g., ref. [26]), consistent with in vitro work [27,28]. In the non-transplant setting, clinicians favor preemptive strategies to combat CMV. For cCMV cases, intravenous ganciclovir and oral valganciclovir are administered during the neonatal period following viral detection [29]. AIDS patients are also treated for CMV preemptively with intravenous ganciclovir and oral valganciclovir, and those who develop CMV retinitis receive intravitreal ganciclovir injections, often coupled with systemic therapy [30]. It is important to note the major limitation of all of these approved therapies is that they fail to target the latent viral reservoir, but are instead only effective against lytically replicating virus when the disease is already primed to occur. Thus, given the toxicity and viral resistance associated with these treatments [19,31] and to prevent HCMV-associated disease prior to on-set, it is crucial to develop novel therapeutics targeting the latent reservoir. 

HCMV is a betaherpesvirus consisting of a 240 kb linear dsDNA genome, the largest of all herpesviruses. The genome consists of distinct regions termed unique long (U_L_) and unique short (U_s_), which are flanked by terminal repeats, terminal repeat long (TR_L_) and terminal repeat short (TR_S_), and internal repeats, internal repeat long (IR_L_) and internal repeat short (IR_s_). Upon infection, this linear genome circularizes and exists as a circular genetic element called an episome within the nucleus of the host cell [32], where it remains tethered to host chromosomes throughout latency [33]. During lytic infection, HCMV, like all herpesviruses, transcribes its genes in a highly coordinated temporal cascade categorized into three stages: immediate-early (IE), early (E), and late (L) [34]. This is a strictly regulated, stepwise process in which IE gene expression relies solely on host transcription factors and viral tegument proteins and thus does not depend on de novo protein translation. IE protein expression is required for activation of E gene expression, whose proteins facilitate viral DNA replication [34]. Following this, L genes are expressed, which primarily encode virion structural components, as well as those proteins necessary for packaging and egress [34]. During lytic infection, this cascade begins with expression from all five sites of IE gene transcription, although regulation of E gene expression is dictated by proteins derived from the major immediate-early (MIE) locus. This region drives expression of the MIE genes, *UL123* and *UL122*, encoding the MIE proteins, IE72 and IE86, respectively. IE86 specifically is required to initiate the subsequent cascade of viral gene expression [35,36,37]. As such, a characteristic of HCMV latency is a significant repression of this locus [38,39,40,41]. Since IE86 transcriptionally activates the E genes, thereby governing the lytic transcriptional profile, regulation at this locus is critical during all phases of infection. Unsurprisingly, mechanisms regulating the MIE locus are multifaceted. While the MIE promoter (MIEP) was originally thought to solely drive transcription of *UL122* and *UL123*, recent work highlights this region is more complicated [42]. The intricacies in the regulation of the MIE locus undoubtedly contribute to and aid in dictating the phase of HCMV infection. Herein, we discuss the complex regulation of the MIE region and how its control impacts viral infection and HCMV pathogenesis.

## 2. Cell Type-Specific Regulation of the MIE Locus

The outcome of HCMV infection is cell type-specific. HCMV lytically infects many cell types including fibroblast, smooth muscle, endothelial, epithelial, terminally differentiated dendritic, and macrophage cells [43]. HCMV resides latently in myeloid lineage cells, CD34^+^ hematopoietic progenitor cells (HPCs), and circulating CD14^+^ monocytes [44,45,46,47,48], and differentiation of these latently-infected cells reactivates HCMV and induces lytic gene expression and virus production [38,48,49,50]. It is important to note that a growing body of literature reinforces the idea that monocytes also support quiescent infection [51,52,53], defined by reactivation in the absence of external activation signals [54,55]. However, as the virus can be reactivated from latently-infected monocytes following growth factor stimuli (e.g., refs. [38,47,50,56,57,58]), these cells are a popular and accepted model of latency. While our understanding of quiescence is still developing, it shares similar characteristics with latency, including transcriptional silencing of lytic gene loci, including the MIE region [59]. Thus, for this review, we will refer to the quiescent infection of monocytes as latent. 

Reactivation of HCMV upon differentiation of myeloid cells suggests coordination of regulation at the MIE locus and myeloid lineage commitment genes. Cell fate and differentiation are tightly regulated by cellular gene expression in the myeloid lineage [60], suggesting the same sets of transcription factors and chromatin-modifying enzymes may be involved. For example, the transcriptional repressors CUX1/CDP (cut like homeobox 1/CCAAT displacement protein) and Growth Factor Independent 1 (Gfi-1) are downregulated during myeloid differentiation, while transcriptional activators PU.1, CCAAT-enhancer-binding protein (C/EBP), activating transcription factor/cyclic AMP response element (CRE)-binding protein ATF/CREB, activator protein-1 (AP-1), and nuclear factor kappa B (NFκB) are upregulated [61]. Further, increased levels of the lineage-determining transcription factor PU.1 are important for differentiation of macrophages and neutrophils [62,63], and this host protein upregulates myeloid-specific cell surface antigens in progenitors while downregulating other cell-specific markers and transcription factors [64]. To date, myeloid-specific transcriptional repressors have not been identified, but this does not exclude the possibility of cofactors or upstream regulators that have a myeloid-specific expression that could contribute to regulating the MIE locus in myeloid cells. Recent genome-wide studies in macrophages and dendritic cells (DCs) revealed the enhancer landscape, defined by mapping histone modifications and a panel of myeloid-specific transcription factors, is unique to the cell lineage and correlates with lineage-specific gene expression [65]. In the context of HCMV infection, this may explain why the virus preferentially infects a subset of myeloid cells. One could hypothesize the transcriptional landscape during latency and reactivation impacts lineage-specific differentiation, directing cells towards the macrophages and DCs, as opposed to the other cell types in the myeloid lineage. Or equally plausible, that differentiation down a different lineage (e.g., lymphoid) results in an abortive reactivation. While several groups have devoted significant efforts towards defining the latent transcriptome in myeloid cells [66,67,68], additional work aimed at linking the transcriptional profile to the epigenetic landscape will help delineate these possibilities.

## 3. Structure of the MIE Locus 

**Figure 1:**
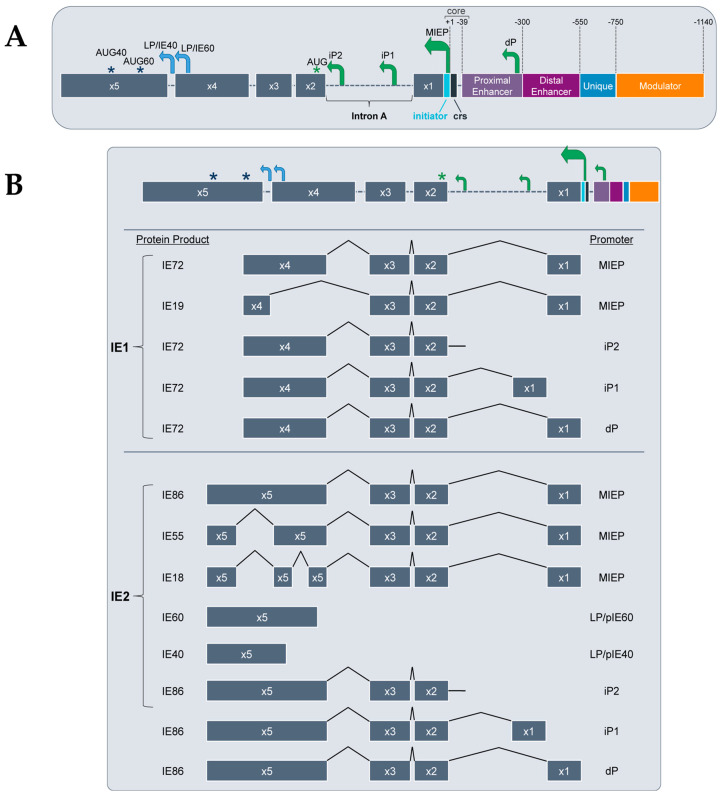
The MIE locus architecture, gene and protein products, and the promoters that drive them. (**A**) The MIE locus consists of a core promoter (−39 to +1), proximal and distal enhancers (−300 to −39 and −550 to −300, respectively, as well as unique (−750 to −550) and modulator (−1140 to −750) regions. Within the core promoter, also known as the MIE promoter (MIEP) lies the cis-repression sequence (crs) from −13 to +1. Alternative promoters include the distal promoter (dP) in the proximal enhancer and the two intronic promoters, iP1 and iP2. Late promoters (LP; blue arrows) upstream of exon 5 drive transcription from start sites within exon 5. With the exception of those derived from the IE40 and IE60 promoters (LP/pIE40 and LP/pIE60, respectively), all transcripts use a common AUG (designated by the green asterisk) in exon 2 (x2). AUG40 and AUG60 (blue asterisks) denote the start sites for IE40 and IE60, respectively. (**B**) Transcripts derived from this locus are alternatively spliced and can be categorized by IE1- or IE2-derived based on the alternative inclusion of exon 4 or exon 5, respectively. The promoters driving each transcript are depicted at right, and the protein products resulting from their translation are depicted at left.

The regulatory region controlling the expression of the IE genes, *UL123* and *UL122,* is comprised of a core promoter, several alternative promoters, an enhancer, a unique region, and a modulator (Figure 1A). The core promoter, or the MIEP, is positioned −39 to +1 nucleotides from the transcription start site and contains a TATA-box between −28 and −22 and a *cis*-repression sequence (crs) between −13 and +1 [69,70,71]. The MIE enhancer is further characterized into the proximal enhancer (between position −299 to −39) and the distal enhancer (between position −579 to −300) [72,73,74]. Most transcription factors that bind to and regulate the MIE locus bind within the enhancer region, although some bind the unique (position −750 to −500) or modulator regions (position −1140 to −750) [75]. The enhancer regulates transcription, in part, through small *cis*-acting repeat sequences of 18-bp, 19-bp, and 21-bp, to which trans-acting factors bind [74,76,77]. The precise functions of the unique and modulator regions in controlling transcription from this locus are largely unknown, although many transcriptional activators and repressors bind each in a cell type-specific fashion [78,79,80]. More recently, Arend, et al. found alternative MIE promoters that are active during lytic infection of fibroblasts. These newly identified alternative promoters are distinct from each other and the MIEP and each produces transcripts encoding the canonical IE72 and IE86. The MIEP produces a transcript with a 5′ untranslated region (UTR) of 136 bp (UTR136), whereas each alternative promoter drives the expression of a distinct transcript varying in 5′UTR length. These alternative promoters include the distal promoter (dP), which produces UTR487, and two intronic promoters, iP1 and iP2, which yield UTR378 and UTR70, respectively [42] (Figure 1B). As iP1 and iP2 are located within intron A, each of their transcripts lack the noncoding exon 1 [42]. In addition to functioning during lytic infection, these promoters are active in myeloid cells under conditions that favor latency, and these promoters play a role in reactivation in myeloid cells [42,81,82,83]. That these alternative promoters were only recently identified suggests our understanding of the MIE locus is far from complete. Indeed, this illuminates the complexity of this locus, as to how it regulates MIE gene products during distinct phases of infection, and ultimately, how this regulation impacts pathogenesis and disease.

## 4. MIE-Encoded Proteins

The MIE locus encodes several proteins produced by alternative splicing of the MIE genes. The MIE gene locus consists of 5 exons and 2 polyadenylation (polyA) signals that give rise to the spliced mRNAs (Figure 1B) [84]. Transcription of *UL123* and *UL122* yields two major protein products, IE72 (often commonly referred to as IE1) and IE86 (commonly termed IE2), respectively, derived from alternatively spliced mRNA transcripts [85,86,87]. In addition to IE72 and IE86, other gene splice variants have been identified including *UL123*-derived IE19 [88] and *UL122*-derived IE55 [89] and IE18 [90]. IE72 is transcribed from full-length exons 1–4, whereas the IE19 splice variant has an internal deletion from Val(86) to Pro(404) in the sequence [88], suggesting the IE72 functional domain lies within this region. A biological role for IE19 in genomic maintenance and subsequent viral production in lytically-infected fibroblasts was recently identified [91]; if this protein is required for viral genome maintenance during latency, however, remains elusive. IE86, the most studied *UL122* gene product, is transcribed from full-length exons 1–3 and 5, whereas IE55 is a splice variant with an internal deletion of 154 amino acids in exon 5, which acts as a transcriptional activator in reporter assays [89]. IE18, a derivative of IE55 containing an additional 325 nucleotide deletion [90], displays cell type-specific expression. In lytically-infected fibroblasts, IE18 was expressed only in the presence of protein synthesis inhibitors, however, IE18 was detected in infected monocyte-derived macrophages [90]. IE40 and IE60 are derived solely from exon 5 [92,93,94,95], and disruption of the sequence of each of their TATA boxes results in attenuated lytic replication [95]. IE40 also functions similarly to IE86, as it too binds the crs site to repress MIEP-driven transcription [95,96,97]. Late in lytic infection of fibroblasts, IE40 and IE60, along with IE86, facilitate transcription from specific viral promoters by enhancing PolII recruitment [98]. As mentioned above, IE86 is the viral protein critical for furthering the viral lytic cycle by transactivating E transcripts, conferring an essential role for this MIE protein in viral replication [99,100]. However, it also autoregulates the activity of the MIE locus by binding the crs site [101], thus preventing RNA pol II binding and in turn repressing the MIEP [69,70,102,103]. Additionally, IE72 and IE86 can each activate cellular promoters, including c-fos, c-myc, and hsp-70 [104,105,106,107], as well as inhibit intrinsic and innate host responses [35,108,109,110,111]. Thus, it is clear IE72 and IE86 have vast and important roles in HCMV gene expression and infection stage, which largely contribute to pathogenesis.

## 5. Roles of Chromatin Structure and Remodeling in MIE Locus Activity 

Gene expression is a complex process that is partially regulated by chromosome organization, which at the smallest unit, is made up of DNA and histones. The negatively charged DNA binds the positively charged amino acids of the histones. The amino-terminal histone tails protrude from the nucleosome core and are then subject to a variety of post-translational modifications (PTMs), predominantly on H3 and H4 histone tails [112]. These histone PTMs, including acetylation, methylation, phosphorylation, ubiquitination, and sumoylation [113], regulate chromatin structure by making contact with adjacent nucleosomes and recruit remodeling enzymes that reposition nucleosomes, thereby making DNA accessible [112]. While the packaged viral DNA within a virion is naked, the HCMV genome is chromatinized upon infection of the host cell [114]. As a region requiring tight transcriptional regulation, the MIE locus is highly regulated by histone PTMs [115]. It is important to note that DNA methylation, an epigenetic modification that regulates gene expression [116], of the MIE enhancer/promoter has been minimally studied. While there is some evidence for MIE locus DNA methylation, these studies were performed in transient expression assays, as opposed to infection [117,118,119,120,121,122,123,124]. To date, data supporting the notion that DNA methylation regulates MIE enhancer/promoter activity is limited. Thus, we will focus this section on our current understanding of histone PTM-mediated chromatin remodeling of the MIE locus.

### 5.1. Repression of the MIE locus by Histone PTMs 

**Figure 2:**
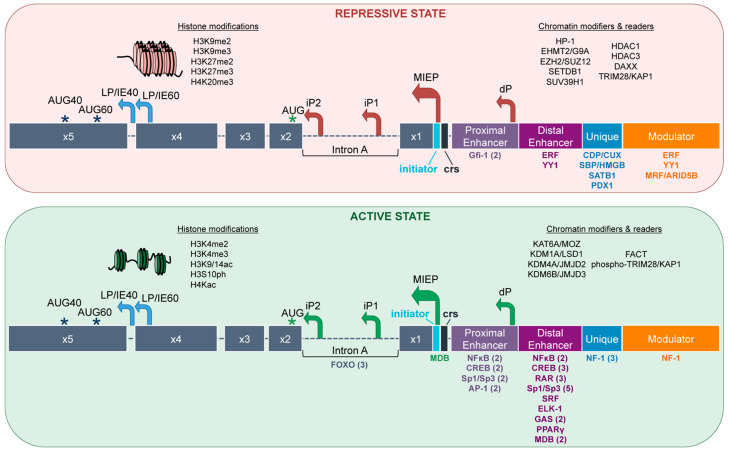
MIE locus architecture during repressive and active states. Histone modifications and chromatin modifiers and readers associate with the entirety of the MIE region. On the other hand, transcription factors are recruited to the enhancer, unique, and modulator regions. Together, these factors regulate the balance of this locus between repressive (**top**) and active (**bottom**) states. During latency and quiescence, the MIE locus is repressed, while reactivation triggers the switch to a more active state, which is maintained during lytic replication. When repressed, the locus is in a closed chromatin conformation (**top**), which is relaxed and opened once activated (**bottom**). Chromatin modifiers and readers are noted for each state at the top right of each box. Transcription factors are shown below the locus and are color-coded based on the region to which they bind. Numbers next to the transcription factors denote the number of sites within that specific region. Those without a number have a single site within the designated region. AUG40 and AUG60 (blue asterisks) denote the start sites for IE40 and IE60, respectively. Canonical AUG noted by green asterisk.

Methylation of histones is associated with transcriptionally inactive regions of the human genome [125]. Specifically, histone H3 di- and tri-methylated (me2 and me3, respectively) modifications at lysine residues 9 and 27 (K9 and K27, respectively) [126,127] and H4K20me3 [48] leads to silencing [128]. Their function is augmented by the recruitment of heterochromatin protein 1 (HP-1) and polycomb proteins to promoters through a high-affinity interaction with H3K9me3 and H3K27me3, respectively [126,129,130]. These histone modifications on the viral genome are also associated with repression of transcription (Figure 2). Indeed, H3K9me2, H3K9me3, and H3K27me3 at the MIE locus correlate with low levels of UL123 and UL122 gene transcription [131,132,133,134,135], suggesting this region is in a closed conformation where binding sites are inaccessible to transcriptional activators and RNA pol II when studded with these histone marks. This closed conformation at the MIE locus, including the presence of HP-1, is synchronous with HCMV latency [48,49,135]. Histone methyltransferases (HMTs) and histone deacetylases (HDACs) work in concert to modify histone PTMs to regulate the MIE locus. HMTs such as EHMT2/G9A, EZH2, SETDB1, SUV39H1 regulate the MIE locus [103,136,137,138,139,140,141,142,143,144,145,146] through the addition of methyl groups that define access to the DNA. As a part of multiprotein complexes, HDACs catalyze the cleavage of acetyl marks from proteins, including histones [147]. As such, HDACs, including HDAC1, HDAC3, and HDAC4 function to repress transcription from the MIE enhancer/promoter [148]. On the other hand, IE72 and IE86 antagonize HDAC1 and HDAC3 to facilitate viral replication [141]. Further, HDACs are likely recruited by transcription factors, which once bound, recruit additional transcription factors [135]. For example, HDAC1 associates with Yin Yang 1 (YY1) [149], a known transcriptional repressor of the MIE locus [150], thereby creating a repressive complex [151]. Collectively, these data suggest repression of the MIE locus via histone PTM is hierarchical. 

### 5.2. Activation of the MIE Locus by Histone PTMs 

Acetylation of histone H3 (K9 and K14) and H4 (K5, K8, K12, and K16) lysine tails is associated with active gene transcription [135,152,153]. Acetylation leads to weaker binding of the nucleosome components, rendering the DNA more accessible to transcription factor binding. Methylation of H3K4 and phosphorylation of serine 10 of H3 (H3S10ph) are also characteristic of transcriptional activation [154,155]. Thus, it is unsurprising that H3K4me2, H3K4me3, H3K9/14ac, H3S10ph, and H4Kac recruitment to the MIE locus results in increased gene transcription from this region (Figure 2) [48,49,131,132,133,134,135,156,157], suggesting these modifications render this enhancer/promoter more accessible to transcription factors that promote active transcription. Histone acetyltransferases (HATs; e.g., KAT6A/MOZ) and demethylases (e.g., KDM1A/LSD1, KDM4A/JMJD2, KDM6B/JMJD3) activate the MIE locus by adding acetyl groups and removing methyl groups from histones at the MIE enhancer region [140], which is necessary for transcription initiation from this locus during reactivation. Further, in differentiated monocyte-derived DCs, which support lytic infection following reactivation, the MIE locus is predominantly studded with acetylated histones, whereas this is not the case in undifferentiated monocytes, where the virus resides latently [48]. Together, these data suggest a closed chromatin conformation at the MIE locus during latency, consistent with genomic silencing, while this region supports an open chromatin conformation following reactivation, indicative of transcriptional activity. The chromatin conformation of a gene’s enhancer/promoter is principal in determining if transcription from that region will occur. In this manner, the MIE locus has tightly packed chromatin and repressive chromatin marks during latency, where transcription from this region is significantly repressed. However, as the virus reactivates, the MIE locus displays a relaxed chromatin configuration with chromatin marks indicative of active gene expression, resulting in the initiation of the temporal cascade of lytic gene transcription.

## 6. Roles of Transcription Factors in MIE Enhancer/Promoter Activity

Transcription factors play a major role in the expression and regulation of genes, and transcriptional control of the MIE locus is no exception. Because expression of the MIE genes is paramount to infection of the host cell, regulation of this region is under tight control. There is a unique balance between activation and repression of this enhancer/promoter region that is partially controlled by cellular transcription factors (Table 1), allowing for distinct gene expression from this locus during lytic infection versus latent infection (Figure 2).

### 6.1. Transcriptional Repressors Bind During Latency

Latency is characterized by transcriptional silencing, which is due in part to the association of repressive transcription factors that work alongside chromatin remodeling factors discussed above. This silencing allows the virus to evade the host immune response while quiescent, thereby securing life-long infection. As with chromatin remodeling, regulation of the MIE locus via transcription factors is a dynamic process that allows the MIE enhancer/promoter to toggle between an active (lytic), repressed (latent), and de-repressed (reactivated) state. 

Most of the transcription factors that bind in the MIE locus do so within the enhancer region (Figure 2). The proximal enhancer is bound by the transcriptional repressor, Gfi-1, a 55-kDa nuclear zinc finger protein that binds DNA in a sequence-specific manner [174]. There are two Gfi-1 binding sites, TAAATCAC(A/T)GCA, found in the MIE locus [182]. Transfection of Gfi-1 into NIH 3T3 fibroblasts repressed the MIEP by chloramphenicol acetyltransferase (CAT) reporter assay, which was reversed by mutation of critical residues in the two Gfi-1 binding sites [182]. However, Gfi-1 alone may not be enough to control the MIEP; during blood cell development, Gfi-1 functions as part of a complex along with other cofactors to control histone modifications that lead to transcriptional silencing [183], which suggests a potential, coordinated approach to silencing the MIE enhancer/promoter. Further, *Gfi-1* mRNA expression is enhanced in the bone marrow and lymphoid tissue and is essential for hematopoiesis [183]. As HCMV resides latently in cells of the hematopoietic lineage, it is attractive to hypothesize a function for this transcription factor in regulating latent infection and reactivation. Future work aimed at understanding Gfi-1′s function during latency, as well as how this transcription factor is controlled in hematopoietic cells, will provide added clarity to the functional regulation of the MIE locus.

The distal enhancer, consisting of 300 bp upstream of the proximal enhancer region, has binding sites for both YY1 and Ets-2 Repressor Factor (ERF) transcription factors. YY1 is a zinc finger DNA-binding transcription factor with the ability to activate, repress, or initiate transcription depending on the promoter. Its function is partially dependent upon different binding partners and the recruitment of varying cofactors of YY1 [149]. YY1 binds the MIE enhancer region at two, 21 bp repeat elements and binds the consensus sequence 5′-CCGCCATNTT-3′, originally shown in undifferentiated non-permissive NTera-2 cells [150]. In cancer cells, YY1 recruits and binds directly to HDACs through bridge proteins to enable transcriptional repression [149]. YY1′s ability to bind varying cofactors in a context-dependent manner suggests this transcription factor plays a critical role in HCMV latency through recruitment of silencing factors and chromatin remodelers, although its precise function during latency remains elusive. YY1 also contributes to enhancer-promoter interactions in mammalian cells and murine embryonic stem cells, similar to DNA looping mediated by CTCF [184]. DNA looping allows for the regulation of promoters by distant enhancers through the interaction of two proteins. CTCF binds within the first intron of the MIE locus, and the deletion of this CTCF binding site leads to an increase in MIE expression and viral replication [175], although a role for CTCF during latency is currently unknown. It is attractive to speculate that this transcription factor, which can act as a barrier protein [185], may help to repress the MIE locus in this manner during latent infection. Therefore, studies aimed at understanding YY1′s contribution to such interactions could provide a better understanding of the MIE locus architecture during latency and reactivation. ERF, another transcriptional repressor, is a nuclear transcription factor that represses MIE enhancer/promoter activity in transient transfection assays in undifferentiated non-permissive NTera-2 cells [176]. As an Ets family transcription factor, ERF binds the consensus DNA sequence 5′-GGA(A/T)-3′, present within the enhancer’s 21 bp repeats and the modulator [186]. ERF also interacts with HDAC1, leading to repression of the MIEP [176] in NTera-2 cells [186]. As YY1 and ERF both repress the MIE locus and have their own consensus sequences within the 21 bp repeat elements of the proximal enhancer, it is possible these transcription factors work in concert to repress transcription from this region. Work aimed at unraveling the contribution of each transcription factor alone and in combination in regulating the MIE enhancer/promoter in the context of latent infection could reveal mechanisms underlying latent control of this region.

Four transcriptional repressors bind the unique region of the MIE locus, including CUX1/CDP [177], HMGB/SBP [178], SATB1 [179], and PDX1 [180]. CUX1/CDP is a member of the conserved Cut/CDP family of homeodomain DNA binding proteins [177]. The MIE unique region contains putative binding sites for CUX1/CDP, and its overexpression results in the inhibition of the MIEP in reporter assays [177]. The binding of this transcription factor prevents the recruitment of positively-activating CCAAT factors to promoters [187], which presumably mediates this observed repression. Thus, in contrast to YY1 and ERF, CUX1/CDP employs a different strategy to silence the MIE locus, suggesting HCMV evolved several means to ensure this region remains silenced throughout latency. Another silencing factor with a distinct function is silencing binding protein (SBP/HMGB), which belongs to the high mobility group box (HMGB) superfamily. SBP/HMGB is a non-histone, nuclear DNA-binding protein that regulates transcription and is involved in the organization of DNA [188]. Hence, HMG proteins are architectural factors that can modulate nucleosome and chromatin structure [189]. A transcriptional repressor with this ability is beneficial to HCMV, as the chromatin structure of the MIE locus needs to be efficiently remodeled to establish latent infection. SBP/HMGB binds in the unique region of the MIE locus [178], which suggests it acts as an insulator between the enhancer region and the adjacent ORF, *UL127*. Similarly, SATB1 (Special AT-Rich Sequence Binding Protein 1) binds the unique region between −593 and −549 [179]. How SATB1 regulates the MIE locus is unclear. However, when SATB1 is bound to DNA, it recruits chromatin remodeling enzymes by associating with HDACs or HATs [190]. Thus, perhaps SATB1 functions to maintain a latent state in myeloid progenitor cells by recruiting chromatin modifying enzymes to the MIE locus to ensure transcriptional silencing of this region. 

Several other host factors have binding sites within the MIE locus that likely contribute to its regulation. Pancreatic duodenal homeobox factor 1 (PDX1) regulates HCMV gene expression by repressing MIE activity via a 45-bp element at position −593 to −549 upstream of the transcription start site [180]. A decrease in MIE transcription by luciferase assay results from the overexpression of PDX1 in 293 cells, and conversely, luciferase expression increases when the PDX1 binding sites in the promoter were mutated or blocked by site-directed mutagenesis [180]. How this cellular protein impacts the MIE locus during lytic or latent infection, however, remains outstanding. Lastly, modulator recognition factor (MRF; also known as ARID5B), binds the MIE modulator [181]. MRF/ARID5B is a member of the AT-rich interaction domain (ARID) family of DNA binding proteins and is thought to play a role in hematopoietic cell development and differentiation [191]. MRF expression is differentiation-specific, as undifferentiated NTera-2 and THP-1 cells display increased protein and mRNA levels, which is reduced following differentiation [181]. This is similar to the facilitates chromatin transcription (FACT) complex protein subunit, suppressor of Ty16 (SPT16; discussed below), whose expression diminishes upon hematopoietic cell differentiation [192]. These transcriptional repressors are only one facet of the complexity of the MIE locus regulation. HCMV latency and reactivation are dynamic processes, and the removal of transcriptional repressors allows for the recruitment of transcriptional activators to the MIE locus, thus leading to activation of the promoters and downstream viral gene expression.

### 6.2. Transcriptional Activators Bind During Reactivation and Lytic Infection

Reactivation from latency is characterized by the de-repression of the MIE locus to initiate lytic gene transcription. While our understanding of the repertoire of factors controlling this is likely incomplete, differentiation of myeloid cells to macrophages or DCs triggers viral reactivation, which in the face of a weakened immune system, leads to lytic replication and viral dissemination [193]. De-repression of the MIE locus requires chromatin remodeling and the exchange of transcriptional activators for repressors. These transcriptional activators have multiple sites within the MIE locus, distributed across the proximal and distal enhancer region (Figure 2). One of the best-studied transcriptional activators of the MIE locus is NFκB, which binds four sites in the proximal and distal enhancers. Indeed, NFκB signaling activates the MIE locus [70,159,162,163,164]. Canonical NFκΒ activation requires phosphorylation by a three-subunit IκΒ kinase (IKK) which subsequently degrades IκΒ, and in the context of HCMV, this can occur as early as 30 min after lytic infection of fibroblasts [164,194]. It is thought this early induction of NFκB helps initiate MIE-driven transcription, in turn accelerating lytic gene expression [162]. While these findings suggest HCMV has evolved to use NFκB to its benefit, the host may also leverage this cellular protein’s functions for antiviral countermeasures. For example, NFκB is an important regulator of pro-inflammatory gene expression by regulating cytokine expression, such as tumor necrosis factor-alpha (TNFα), interleukin-1β (IL-1β), IL-6, IL-8, and cyclooxygenase 2 (Cox-2). To counter these measures, HCMV encodes proteins, including IE86, to target this pro-inflammatory response [195,196,197]. IE86 blocks NFκB signaling, and therefore of IFNβ production, by binding the IFN promoter [195]. Such host measures and viral countermeasures are also evident during latency and reactivation. The aforementioned host-encoded proinflammatory genes also provide reactivation cues and some are used to stimulate reactivation in in vitro settings [48,49,198,199]. 

In addition to NFκB binding sites, ATF/CREB also binds the MIE proximal and distal enhancer, with an additional binding site in the core promoter. There are a total of five CRE sites in this locus [158], characterized by *cis*-acting 19 bp repeats. Mutation of the CRE element at position −137 significantly decreases viral gene expression measured by a CAT reporter assay in primary fibroblast, primary aortic endothelial, telomerase-immortalized retinal pigmented epithelial, and primary hepatocyte cells [200], suggesting CREB binding at this site is important for efficient transcription from this locus. CREB is a phosphorylation-dependent transcription factor [201], binding to the MIE enhancer/promoter is crucial for the induction of both *UL123* and *UL122* expression in reactivating CD14^+^ monocyte-derived DCs [144], which implicates that this transcription factor is important for viral reactivation. In this context, binding of phosphorylated CREB to the MIE enhancer/promoter recruits the mitogen and stress-activated kinase (MSK), which is activated via MAPK signaling. CREB-mediated recruitment of activated MSK promotes histone H3 phosphorylation and subsequent histone demethylation, thereby further contributing to viral reactivation [144]. Collectively, these data strongly support a role for CREB during reactivation, though it remains unclear as to how CREB is activated by upstream signaling and which binding sites of the five are critical for this phase of infection; whether the multiple CREB binding sites work cooperatively or whether they are redundant is unknown. One can envision the importance of multiple CREB binding sites, which collectively may work in concert to recruit sufficient phosphorylated MSK for its functions in this process. Alternatively, perhaps the virus has evolved multiple CREB binding sites to differentially regulate the MIE promoters in a cell-type-specific manner. Future experiments aimed at dissecting such nuances will undoubtedly provide clarity and better our understanding of how this important transcription factor is regulated.

The MIE enhancer region also contains binding sites for ligand-inducible transcription factors, retinoic acid receptors (RAR), and the retinoid-X receptor (RXR-A), both of whose ligand is retinoic acid (RA) [165]. RAR and RXR-A interact with *cis*-acting DNA binding sites called the RA-responsive elements (RAREs) [202,203,204]. RAR and RXR-A heterodimers bind preferentially to RAREs consisting of two direct repeats with the following sequence: 5′-AGGTCA-3′. These direct repeats contain inter-half-site spacing of 1–5 bp (known as DR1–DR5) [205]. Within the MIE enhancer are three RAREs, one DR1, and two DR5 motifs to which RAR and RXR-A bind cooperatively [202,203,204]. Treatment with RA increased IE72 protein expression in NTera-2 cells [206]. In murine CMV (MCMV)-infected murine fibroblasts, treatment with RA increased murine MIE enhancer activity viral growth, and in line with this, RA administration to MCMV-infected mice worsened acute infection [207]. It is important to note, however, the murine and human MIE loci are quite different [208], making it difficult to extrapolate these findings to HCMV without further experimentation. Interestingly, RA plays a role in hematopoietic stem cell differentiation and self-renewal, as loss of RARs leads to reduced numbers of these cells [209]. Given the differentiation of infected hematopoietic stem cells leads to viral reactivation, it is possible RAR may function during HCMV reactivation as myeloid cells differentiate.

There are seven predicted Specificity Protein 1 (Sp1) binding sites in the MIE enhancer [166]. Two of these sites (5′-(G/T)GGGCGG(G/A)(G/A)(C/T)-3′; GC boxes), which bind Sp1 and Sp3 are contained within the minimal enhancer element for the MIE locus at approximately positions −75 and −55 relative to the transcription start site (+1) in the proximal enhancer [73,167]. Sp1 is a transcription factor involved in the activation of a large number of genes necessary for cellular processes such as cell growth, apoptosis, differentiation, and immune responses [210]. Although a distinct protein, Sp3 acts as a transcriptional activator at Sp1-like sites on many promoters [211,212,213]. Sp1 binding to the MIE locus at position −55 increases transcription ~3-fold by CAT reporter assay and binding at both sites bolsters MIE transcription ~35-fold, showing a synergistic effect between these two binding sites. Additionally, mutation of both Sp1 and Sp3 binding sites in the proximal enhancer causes inefficient MIE transcription and viral replication in lytically-infected fibroblasts [167], suggesting redundancy for these proteins in the MIE enhancer during lytic replication. To date, whether Sp1 and/or Sp3 aid in transactivation of the MIE locus during reactivation remains elusive, as direct binding of either transcription factor during any phase of infection has not yet been shown. While these transcription factors are redundant in lytically infected fibroblasts, perhaps they have cell- or tissue type-specific functions. 

The AP-1 transcription factor binds two sites in the MIE proximal enhancer [159,160]. This transcription factor is comprised of c-fos and c-jun heterodimers that activate transcription at 12-*O*-tetradecanoylphorbol-13-acetate (TPA) response elements (TRE) with the consensus sequence 5′-TGAC/GTCA-3′ [160]. The AP-1 consensus site is between positions −174 and −168, relative to the transcription start site of the core promoter at position +1 [214], while the non-consensus site (5′-TGACTAA-3′) is located between positions −239 and −233 [159,160,215]. While neither site alone or in combination is required for efficient lytic replication in fibroblast or epithelial cells [83,160], AP-1 binding to its consensus sequence in the MIE enhancer is critical for successful reactivation from latency, as it is necessary for de-repression of MIE-driven transcripts, but not other IE genes [83]. Additionally, AP-1 recruitment to its consensus site in the MIE enhancer was necessary for the expression of MIEP-, iP2-, and dP-derived transcripts, while iP1-driven transcription was not altered with disruption of the AP-1 canonical binding site [83], suggesting the MIE canonical and alternative promoters differentially respond to transcription factor binding. In agreement with this, Hale et al. recently identified forkhead box (FOX) binding sites in MIE intron A, capable of supporting FOXO1 and FOXO3a binding [161]. These sites are distinct from the FOXA1 binding site in the MIE unique region, which regulates *UL127* and does not have an effect on MIE-driven genes [216]. Indeed, FOXO1 and FOXO3a each initiate transcription from the alternative internal promoter, iP2, and to a lesser extent iP1, in luciferase reporter assays. Importantly, mutation of the three FOXO binding sites in Intron A imparts a significant impairment in the ability of this virus to reactivate when compared to wild type infected CD34^+^ HPCs [161]. FOX transcription factors are also pioneer factors [217], characterized by their ability to directly bind closed chromatin and remodeling it to an open state to facilitate the binding of other transcription factors, thereby altering the chromatin landscape [218]. This is counter to other transcription factors, which only bind available (or open) DNA sequences. It is therefore attractive to hypothesize the FOXO transcription factors assist in making the chromatin at the MIE locus more accessible during reactivation by priming this region for transcriptional transactivation. Nonetheless, it is clear from these recent findings that the multitude of factors recruited to the MIE locus may indeed regulate specific promoters in this region. This could be specific to the phase of infection, the cell type, tissue type, and/or disease state. 

Multiple transcriptional activators bind sites only within the MIE distal enhancer. Two of these transcriptional activators include Serum Response Factor (SRF) and Ets transcription factor/ETS-like kinase-1 (ELK-1) [219]. ELK-1 is part of the Ets family of transcription factors and ternary complex factor (TCF) subfamily, as it forms a ternary complex with SRF at the serum response element (SRE) [220]. SRF and ELK-1 expression are each required for optimal *UL123* and *UL122* gene expression in lytically infected, fibroblasts [159], suggesting they are transcriptional activators of the MIE locus. ERK/MAPK signaling phosphorylates ELK-1, resulting in the regulation of chromatin remodeling through ELK-1’s interaction with CREB binding protein (CBP) [221], which contains intrinsic HAT activity [220]. This suggests a potential role for ELK-1 in reactivation following stimuli activating the ERK/MAPK signaling pathway. ERK/MAPK also regulates immune signaling pathways and is a negative regulator of interferon-gamma (IFNγ) [222]. An IFNγ response element, gamma-interferon activated sequence (GAS) appears twice in the MIE distal enhancer [168]. The deletion of these elements leads to reduced expression of IE72 and IE86 proteins at low MOI, concomitant with reduced viral growth in lytically-infected fibroblasts. While IFNγ treatment stimulated IE72 expression in infected fibroblasts, cells infected with the GAS-deletion virus failed to upregulate IE72 in response to IFNγ, suggesting IFNγ stimulation of the MIE enhancer region activates MIE expression [223]. However, IFNγ has diverse biological functions, including macrophage activation, as well as neutrophil and natural killer cell stimulation during an inflammatory response [224]. Upon IFNγ stimulation of the cell, the JAK-STAT signaling pathway is activated, leading to STAT1 binding to the GAS element [225]. Thus, it is attractive to speculate that in the context of HCMV infection, GAS binding the MIE distal enhancer could stimulate transcription from this locus, thereby contributing to activation of lytic gene transcription. 

Less well-studied transcription factors that activate the MIE locus include nuclear factor-1/CAAT box-binding transcription factor (NF-1/CTF), peroxisome proliferator-activated receptor-gamma (PPARγ), and methylated DNA-binding protein family (MDBP). NF-1/CTF has two sites in the unique and modulator regions of the MIE locus [170,171] and can function as a barrier that can prevent the propagation of repressive chromatin structure [226]. Barrier proteins, in general, are thought to create and maintain adjacent chromatin domains [227], which one can imagine is important for HCMV during reactivation and lytic infection when the virus must prevent the spread of repressive chromatin into the MIE enhancer region. It is therefore not surprising these sites are found in both the unique and modulator regions, as factors that bind these regions function to retain the chromatin in an open and active state allowing recruitment of transcriptional activators. PPARγ is a nuclear receptor that binds DNA to activate gene expression via peroxisome proliferator-responsive elements (PPREs) [228]. The MIE distal enhancer contains PPREs to which PPARγ binds, and this in turn promotes lytic replication in trophoblasts [169]. PPARγ is generally accepted as an anti-inflammatory molecule based on its ability to inhibit cytokine transcription [228]. One can envision taking advantage of anti-inflammatory molecules to modulate MIE-driven transcription to benefit the virus by evading the host cells’ immune response. Another understudied transcription factor is MDBP, a member of a family of ubiquitously expressed proteins [172]. There are three MDBP binding sites: two high-affinity sites in the enhancer and one low-affinity binding site 5bp upstream of the transcription start site [172,173]. While MDBP binding contributes to MIE enhancer activity by CAT reporter assay [229], further studies aimed at understanding its contribution to reactivation and lytic infection are warranted. Whether the functions of these less-studied transcription factors are restricted to lytic replication is currently unknown, but it is certainly plausible they each may contribute to transactivation of the MIE locus during reactivation.

## 7. microRNA Regulation of the MIE Locus 

microRNAs (miRNAs) are non-coding RNAs that regulate protein expression through interaction primarily with the 3′ or 5′ untranslated region (UTR), where the net effect is the inhibition of the target gene’s translation [230]. Many viruses exploit such post-transcriptional regulation by co-opting cellular miRNAs to their own advantage and/or encoding their own miRNAs. HCMV-encoded miRNAs target both cellular and viral genes [231,232,233,234,235], some of which play a role in latency/reactivation by regulating the MIE locus. For example, HCMV miRNA cmv-miR-UL112-1 targets the 3′ UTR of IE72, as demonstrated by luciferase reporter assays [235,236], resulting in downregulation of this protein in lytically infected fibroblasts [235]. When this was discovered in 2008, the authors hypothesized cmv-miR-UL112-1 might function to repress IE72 during latent infection [235], which was subsequently confirmed in primary monocytes [237]. Further, cmv-miR-UL112-1 repression of IE72 in latently infected monocytes functions to prevent IE-specific cytotoxic T cells from recognizing and targeting the latently infected cells [237], suggesting this HCMV-encoded miRNA additionally functions to evade the host immune response. Highlighting the importance of regulating IE72 expression, two other HCMV miRNAs target this protein. Ectopic expression of either cmv-miR-UL25-1 or cmv-miR-UL25-2 in fibroblasts followed by lytic infection results in a reduction in IE72 protein levels, although its expression is not completely abrogated [238]. While it is unclear if either of these cmv-miRs directly target IE72, their overexpression in the context of lytic infection leads to a significant reduction in viral titers [238], suggesting these two viral encoded miRs may target either directly or indirectly the MIE proteins to regulate the lytic cascade. How these cmv-miRs regulate latency and/or reactivation remains elusive, but it is plausible they may function during these phases of infection. Other cmv-miRs have functional roles during latency and reactivation [239]. While cmv-miR-US22 and cmv-miR-UL22A do not directly exert effects on the MIE enhancer/promoter, they influence HPC proliferation and differentiation [240], as well as myelosuppression and viral genomic maintenance [241], respectively.

In addition to the virally-encoded miRNAs, HCMV co-opts cellular miRNAs to its advantage during latency. The hsa-miR-200 cluster specifically targets the 3′ UTR of *UL122*, and when overexpressed in lytically infected fibroblasts, results in IE86 downregulation. Furthermore, when the hsa-miR-200 cluster cis site within the IE86 3′UTR was mutated, this virus failed to maintain latency in primary CD34^+^ cells, suggesting HCMV uses this host miRNA cluster to effectively “mop up” any aberrant transcription from the MIE locus during latent infection [242]. In support of this, miRNA expression patterns often change during cell differentiation [243], thus it is not surprising hsa-miR-200 levels are high in cells that support HCMV latency but are significantly decreased in more differentiated cells permissive for lytic infection [242]. Finally, TGF-β secretion from latently-infected monocytes increases HDAC4 expression via has-miR206, which promotes deacetylation and MIE enhancer/promoter suppression [148]. Collectively, these data show HCMV utilizes host as well as its own miRNAs as an additional layer of regulation during latency and reactivation. 

## 8. Cellular and Viral Factors Involved in Regulating the MIE Locus 

It is clear from our discussion above that a complex balance of transcription factors and chromatinization tightly regulates the MIE locus. These players and their regulators, therefore, help to control the stage of HCMV infection. The transcription factors and chromatin remodeling enzymes are themselves regulated by both viral and cellular proteins to orchestrate HCMV latent and lytic infection (Figure 2). Upon HCMV lytic infection, the MIE locus is subject to transcriptional repression by cellular Death Domain Associated Protein (Daxx), a component of the promyelocytic leukemia nuclear body (PML-NB), which silences viral gene expression through histone deacetylases [244,245]. However, the HCMV virion tegument protein, pp71, degrades Daxx [246], inactivating its repressive function and therefore initiating lytic replication via trafficking to nuclear domains 10 (ND10) in the nucleus upon viral entry [247]. Daxx also functions in latent infection; its knockdown in undifferentiated myeloid cells leads to productive gene expression [248,249]. Conversely, Daxx associates with HDACs to promote transcriptional repression [250,251]. Other components of PML-NBs, such as alpha-thalassemia/mental retardation syndrome X-linked protein (ATRX), PML protein, and SP100 nuclear antigen modify chromatin and act as repressors of MIE gene expression [252,253]. These proteins do not directly bind the MIE locus, but rather they impart their effects through upstream control. Furthermore, recent findings suggest HCMV reorganizes inactive histones and cellular DNA, thereby isolating the viral genome to optimize viral gene transcription and replication [254]. Collectively, these suggest the cellular environment plays a large role in controlling MIE-mediated transcription and therefore the stage of infection. This is likely due to the tightly intertwined mechanisms of the infection stages, as well as hematopoietic cell differentiation. Other cellular proteins implicated in MIE locus repression during latency include the FACT complex and KRAB-associated protein 1 (KAP1). The FACT complex is a heterodimer of SPT16 and structure-specific recognition protein 1 (SSRP1), possessing chaperone protein functions that destabilize nucleosomes by aiding in the separation of histone H2A/H2B dimer in nucleosomes, thereby enabling transcription [255,256]. FACT binds the MIE enhancer/promoter during latent infection, and shRNA-mediated suppression of SPT16 or treatment with curaxins to inhibit FACT leads to decreased *UL123* expression when induced with TNFα to stimulate reactivation [192], suggesting FACT aids in transactivation of this locus in response to reactivation cues. KAP1, which coordinates chromatin-remodeling proteins such as Mi2α, SETDB1, and HP1 [257,258,259], recruits the H3K9 methyltransferase, SETDB1, resulting in the deposition of repressive H3K9me3 histone marks at the MIE enhancer/promoter during latent infection [136]. Furthermore, activation of KAP1 via ATM-mediated phosphorylation induces viral reactivation in CD34^+^ HPCs, which is further bolstered by TNFα treatment [136]. Although the biological mechanism(s) underlying KAP1 signaling remains elusive, these findings suggest KAP1 activity aids in the transition between HCMV latent and lytic infection. 

In addition to the host encoded regulators of the MIE locus, HCMV encodes proteins to modulate this region during latency, including UL138 [260,261,262] and US28 [263,264,265,266,267,268]. UL138, a gene encoded within the ULb’ region of the HCMV genome, enhances H3K9 methylation at the MIE enhancer/promoter during latent infection and represses MIE transcription in THP-1 cells by blocking histone lysine-demethylase activity [142] and regulating EGFR signaling [269,270,271]. The virally-encoded G protein-coupled receptor (GPCR), US28 is required for the establishment and maintenance of latency [263,264,268,272,273]. While the complete repertoire of pathways US28 modulates during latency is likely incomplete, several signaling cascades are known targets of US28 [264,268,272,274], including STAT3 [268], fos/AP-1 [272], NFκB, and MAPK [264]. As NFκB and MAPK each activate the MIE locus in the context of reporter assays or lytic infection [159,162,163,164,275,276], it is attractive to speculate their activity contributes to regulating this region during latency and reactivation. Thus, their suppression by US28 during latency may aid in silencing the MIE locus. Additionally, latent expression of US28 attenuates c-fos [272], a component of the AP-1 transcription factor that activates the MIE locus [160]. Thus, US28-mediated downregulation of c-fos prevents AP-1 binding to the MIE enhancer, suppressing transcription from this region [272]. As mentioned above, AP-1 is critical to the balance between latency and reactivation, as it is required for successful viral reactivation [83]. Thus, US28 likely prevents premature AP-1 binding to the MIE enhancer during latent infection in the absence of reactivation cues. How AP-1 overcomes US28 upon reactivation is currently unknown, though this is likely an important facet in the switch between latency and reactivation. 

Another virally encoded regulator is the long non-coding (lnc) RNA 4.9, which binds the MIE locus and recruits the repressor complex, PRC2 [137], a methyltransferase with activity toward H3K27 in latently-infected CD14^+^ monocytes [277]. This recruitment of PRC2 suggests lncRNA 4.9 acts to suppress *UL123* and *UL122* gene transcription. Further, lncRNA 4.9 plays a role in viral DNA replication through activity at the HCMV origin of replication (*oriLyt*), as knockdown inhibits viral DNA replication and viral growth in HCMV infected fibroblasts [278]. The ability of viral proteins to regulate the other viral proteins as well as cellular proteins necessary for controlling the MIE highlights the extensive co-evolution between HCMV and its human host.

## 9. Conclusions

Strict regulation of the MIE locus through chromatin remodeling and transcription factor binding is necessary to balance latent versus lytic infection. As the “molecular switch” dictating the “on/off” state of the virus, it is not surprising there are a multitude of host and viral proteins regulating this region. Continued research aimed at understanding a complete repertoire of these factors, as well as their upstream regulators, will further elucidate the mechanisms underlying HCMV latency and reactivation.

## Figures and Tables

**Table 1:** Summary of transcription factors that regulate MIE enhancer/promoter activity.

	**Reactivation from Latent Infection ^1^**	**Lytic Infection ^1^**	**Transient Transfection ^1^**
Activating Factors	ATF/CREB [158] AP-1 [83,159,160] FOXO3a [161]	NFκB [70,159,162,163,164] RAR/RXR [165] Sp1/Sp3 [73,166,167] SRF [159] ELK-1 [159] GAS [168] PPARγ [169]	ATF/CREB [158] RAR/RXR [165] Sp1/Sp3 [73,166,167] FOXO1/FOXO3a [161] NF-1/CTF [170,171] MDBP [172,173]
Repressive Factors			Gfi-1 [174] YY1 [150] CTCF [175] ERF [176] CUX1/CDP [177] HMGB/SBP [178] SATB1 [179] PDX1 [180] MRF/ARID5B [181]

^1^ Context in which the data for each transcription factor was shown. To date, functional analyses for the repressive transcription factors have not been evaluated in latent infection models.
